# Predicting Inactive Conformations of Protein Kinases Using Active Structures: Conformational Selection of Type-II Inhibitors

**DOI:** 10.1371/journal.pone.0022644

**Published:** 2011-07-27

**Authors:** Min Xu, Lu Yu, Bo Wan, Long Yu, Qiang Huang

**Affiliations:** State Key Laboratory of Genetic Engineering, School of Life Sciences, Fudan University, Shanghai, China; Institut Pasteur, France

## Abstract

Protein kinases have been found to possess two characteristic conformations in their activation-loops: the active DFG-in conformation and the inactive DFG-out conformation. Recently, it has been very interesting to develop type-II inhibitors which target the DFG-out conformation and are more specific than the type-I inhibitors binding to the active DFG-in conformation. However, solving crystal structures of kinases with the DFG-out conformation remains a challenge, and this seriously hampers the application of the structure-based approaches in development of novel type-II inhibitors. To overcome this limitation, here we present a computational approach for predicting the DFG-out inactive conformation using the DFG-in active structures, and develop related conformational selection protocols for the uses of the predicted DFG-out models in the binding pose prediction and virtual screening of type-II ligands. With the DFG-out models, we predicted the binding poses for known type-II inhibitors, and the results were found in good agreement with the X-ray crystal structures. We also tested the abilities of the DFG-out models to recognize their specific type-II inhibitors by screening a database of small molecules. The AUC (area under curve) results indicated that the predicted DFG-out models were selective toward their specific type-II inhibitors. Therefore, the computational approach and protocols presented in this study are very promising for the structure-based design and screening of novel type-II kinase inhibitors.

## Introduction

Human genome contains about 518 genes which encode protein kinases (PKs) and account for approximately 2% of the whole human genes [1]. This large protein family is responsible for regulating nearly every aspect of the cellular activities through protein phosphorylation. And unregulated PK activities often cause severe human diseases, such as cancers, inflammation and neuronal disorders etc. [2], [3]. Indeed, the PK catalytic domains are one of the most common domains in which mutations may lead to human cancers. For such reasons, protein kinases have long been regarded as one of the most important families of drug targets [4], [5], [6].

Although the number of human PK family members is large, the existing X-ray crystallographic structures showed that the three-dimensional (3D) structures of their catalytic domains are similar [7]. Typically, the catalytic domain of a PK consists of a smaller N-terminal lobe (N-lobe) and a bigger C-terminal lobe (C-lobe) [8]. And the ATP-binding site is located in a deep cleft between these two lobes. The catalytic residues and the activation loop that are crucial for phosphoryl transfer reaction are located in the cleft. Almost in all PKs, at the N-terminal of the flexible activation-loop there exists a conserved three-residue motif, Asp-Phe-Gly (DFG). The conformational state of this motif has been shown to be a determining factor to the PK activation [9], [10]. In the active state, the phenylalanine (Phe) side-chain occupies the ATP-binding pocket, and the aspartate (Asp) side-chain is located in the outside of the pocket (DFG-in conformation). When the so-called ‘DFG-flip’ occurs, the Asp and Phe residues swap their positions: the Asp side-chain rotates into the ATP-binding pocket, and the Phe side-chain rotates out of the ATP-binding pocket (DFG-out conformation), leading the PK to the inactive state [10], [11]. Some human kinases were shown to be able to adopt the DFG-out conformation [12], [13], [14], and it was suggested that the DFG-in and DFG-out conformations might actually co-exist in the way of dynamic equilibrium [10].

Since a PK in the DFG-out conformation is inactive, it is very interesting to develop inhibitors to specifically recognize the DFG-out conformation [15], [16]. Several inhibitors have already been found to be able to bind to and stabilize the DFG-out inactive forms of their kinase targets [17], [18]. They have been shown to be more specific and effective than those inhibitors which target the active DFG-in conformation (i.e., type-I inhibitors) and therefore were called type-II inhibitors [19], [20], [21]. One example is the anti-cancer drug imatinib (Gleevec, Novartis), which specifically binds to the DFG-out conformations of the tyrosine kinases BCR-ABL, c-Abl, c-Kit and PDGFR [22], [23], [24], [25]. And, as known, structure-based drug design is a very important approach to the discovery of novel type-II kinase inhibitors [16], [26]. However, so far only a few kinase DFG-out structures have been solved, and the structural information about the DFG-out conformations for a large number of kinases is still lacking [27]. Currently, in the Protein Data Bank (PDB), more than 70% of mammal kinase structures are in the DFG-in conformation, and 22% are intermediate structures, about 3% are apo-DFG-out structures which are type-II incompatible [28]. This certainly poses a difficult problem for employing the structure-based design approaches to the discovery of novel type-II kinase inhibitors, because in this approach the kinase DFG-out structures are one of the prerequisites. To address this, it is necessary to develop computational methods which are able to predict DFG-out structures using the large numbers of the existing DFG-in structures.

Recently, Kufareva and Abagyan have already developed a computational protocol for converting DFG-in structures of various kinases into type-II bound state by deleting about six residues of the activation-loop starting with the DFG-motif, i.e., the so-called DOLPHIN (deletion-of-loop Asp-Phe-Gly-in) models [28]. The DOLPHIN models suggested that the main factor affecting the binding of type-II inhibitors could be attributed to the difference of the DFG motif and its neighbor residues between DFG-in and DFG-out conformations. Inspired by this study, here we present a new computational approach called activation-loop remodeling method (ALRM) to predict the DFG-out inactive conformations using the DFG-in active structures of PKs. To the end, we used the DFG-in structure of a protein kinase as the starting template, and employed the protein modeling program Rosetta [29] to predict a large number of possible lowest-energy conformations for its activation-loop beginning with the DFG motif, and then selected appropriate DFG-out models according to the space of the active-site cleft. Moreover, because in the process of the DFG-flip, significantly conformational changes not only occur in the activation-loop, but also in the N-lobe, especially in helix αC [10], [11], [30], to mimic such conformational change, the N-lobes of some DFG-in kinases were rotated about a pre-defined axis before the phase of the activation-loop remodeling, and the rotational angles were determined by the criteria obtained by the analysis of the existing kinase structures. To test the quality of the obtained DFG-out models, we predicted the binding modes for the known type-II inhibitors (Table 1) based on these models, and the results were in very good agreement with the X-ray crystal structures. Also, we tested the abilities of these models to recognize their corresponding type-II inhibitors by screening a small-molecular database which contains about 750 protein inhibitors, including the known type-II inhibitors (see Table S1 in Supporting Information). Results showed that the predicted DFG-out models were selective toward their specific type-II inhibitors. All these results suggested that the presented computational approach would have practical applications in the structure-based design and screening of novel type-II kinase inhibitors.

**Table 1:** DFG-in structures of protein kinases for DFG-out conformation prediction.

Kinases	PDB codes of DFG-in structures and chain IDs	PDB codes of type-II inhibitors and corresponding PDB codes of kinases in the DFG-out conformation
ABL1	2F4J(A)	406(2E2B), 7MP(2HIW), <2?show=[sr]?>GIN(2HZ0), KIN(2HZN), PRC(1FPU), STI(1IEP, 1OPJ, 2HYY)
BRAF1	2FB8(A,B)	BAX(1UWH, 1UWJ)
EPHA3	2QOQ(A)	IFC(3DZQ)
KIT	1PKG(A,B)	STI(1T46)
LCK	3LCK(A)	1N8(2OG8), 242(2OFV), 9NH(3B2W), STI(2PL0)
MK14	1M7Q(A)	1PP(2BAJ), AQZ(2BAK), B96(1KV2), BMU(1KV1), L09(1WBN), L10(1W82), L11(1W83), LI2(1WBS), LI3(1WBV), WBT(1WBT)
SRC	1Y57(A)	STI(2OIQ)

## Results and Discussion

### Outward movements of N-lobes in DFG-out structures

**Figure 1:**
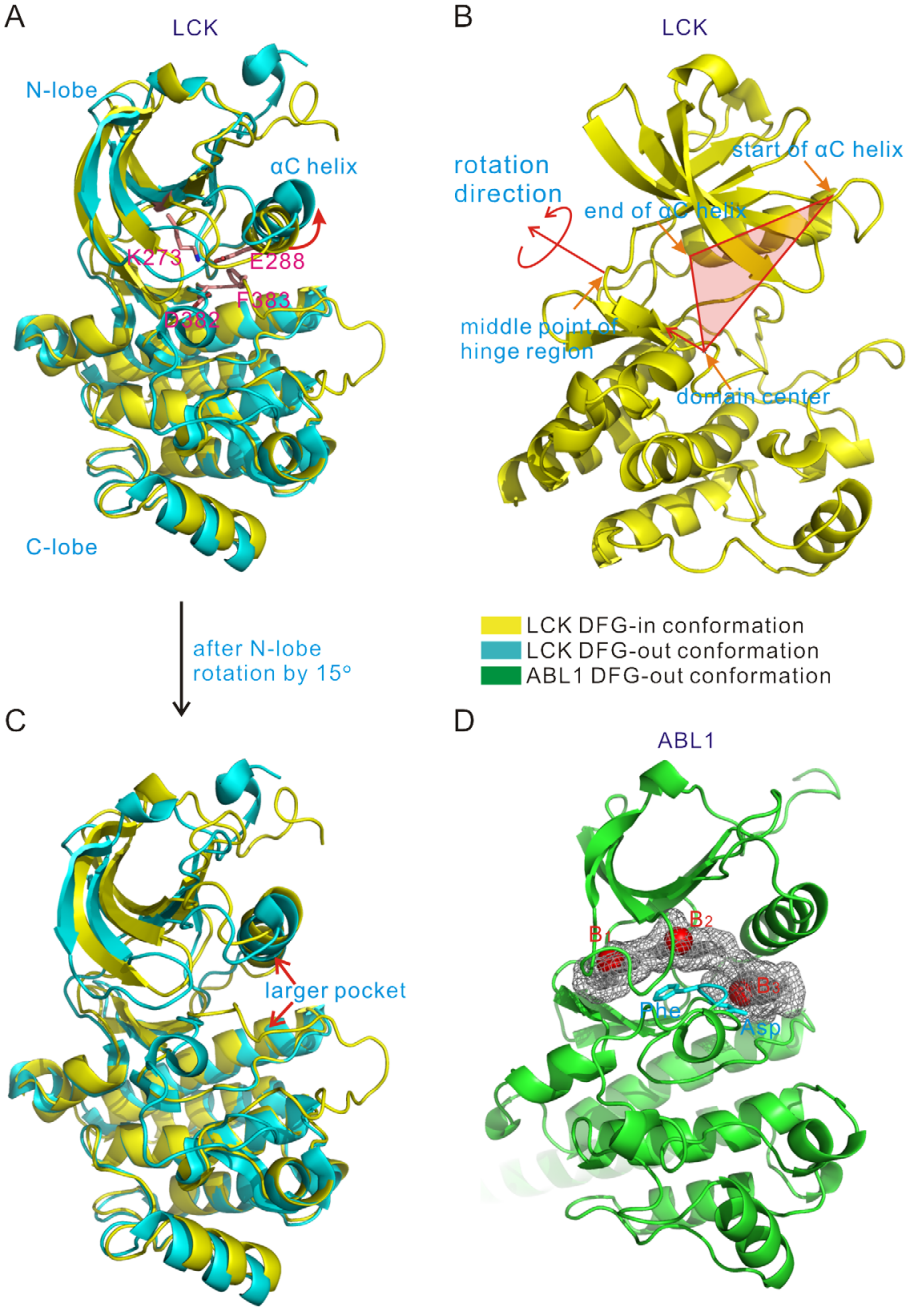
Analyses of kinase DFG-out structures. (A) Superposition of the DFG-in and DFG-out structures of LCK (PDB codes: 3LCK and 2PL0, respectively), and the conserved residues that characterize the space of the active-site cleft (LCK numbering: K273, E288, D382, and F383). (B) Rotation axis of the N-lobe shown by the LCK DFG-in structure. (C) Superposition of the LCK DFG-out structure and the DFG-in structure with the rotated N-lobe. (D) Analyses of the active-site cleft of the kinase ABL1 DFG-out structure (PDB code: 2F4J) using the programs PASS and LIGSITE. The three red spheres B_1_, B_2_, and B_3_ represent the three binding site centers identified by PASS. The grid points enclosed with the gray mesh represent the cleft binding pocket identified by LIGSITE.

We searched the Protein Dada Bank (PDB) at the beginning of this study and found seven kinases with both the DFG-in and DFG-out structures, as listed in Table 1. To prepare the starting all-atom structures for the structural prediction afterwards, we used the program Rosetta to relax the obtained X-ray crystal DFG-in structures. Then we aligned these relaxed structures with the DFG-out structures and found that the N-lobe conformations in the DFG-out structures of some kinases are different from their corresponding conformations in the DFG-in structures. As illustrated in Fig. 1A, the N-lobe of the LCK DFG-out structure moves more outward than that of the relaxed DFG-in structure, and leads to a wider active-site cleft. It appears that in these kinases a sufficiently wide pocket between the N-lobe and C-lobe is required for the DFG-flip and the binding of the type-II ligands [10], [11], [16]. In contrast, in the crystal DFG-in structures, part of the N-lobe such as the helix αC usually moves inward to the active-site cleft to form a more compact conformation and leads to a narrow cleft.

To deeply understand the differences between the DFG-out and DFG-in conformations, we further examined the outward movements of the N-lobes in the mentioned structures and found that such structural changes could be attributed to rotations of the N-lobes around an axis. As indicated in Fig. 1B, the rotation axis is perpendicular to the plane which is defined by the center of the kinase catalytic domain and the start and end residues of the helix αC, and roughly passes through the residue in the middle of the hinge region which links the N-lobe to the C-lobe. With respect to the relaxed DFG-in structures, the N-lobes in the DFG-out structures rotate around the defined axis about 5∼15 degrees (Table 1). To quantitatively characterize the space of the active-site cleft induced by the N-lobe rotations, we used the sum of 4 pairwise distances among four conserved residues. These four residues are Lys273 (LCK numbering, see PDB code: 3LCK) of the β-sheet and Glu288 (LCK numbering) of the helix αC, which form a conserved salt-bridge, and Asp and Phe of the DFG-motif (see Fig. 1A). And the 4 pairwise distances are those from Lys273 to Asp, Lys273 to Phe, Glu288 to Asp, and Glu288 to Phe, respectively. If the sum of these 4 distances is less than 30 Å, the cleft space is considered to be too small; the space is thought large enough if the sum is greater than 32 Å; and the value between 30 and 32 Å suggests that the cleft just needs a slight enlargement. Based on the above observations, we divided the N-lobe rotations of DFG-in structures into three categories: no rotation for the sum greater than 32 Å, rotations by 5 degrees for the distance sum between 30 Å and 32 Å, and 15 degrees for the sum less than 30 Å (Table 2). And after the rotation, the N-lobe conformations of the starting structures are very similar to those in the DFG-out structures with type-II ligands, as illustrated in Fig. 1C.

**Table 2:** Three categories of the N-lobe rotations in the DFG-in structures.

Rotation states	Kinases	Distance sum of four conserved residues (Å)
No rotation (D>32 Å)	ABL1	32.8
	MK14	36.7
5 degrees (30≤D≤32 Å)	EPHA3	30.5
	SRC	30.9
15 degrees (D<30 Å)	BRAF1	25.7
	KIT	22.6
	LCK	28.5

To further analyze the volumes of the active-site clefts in the known DFG-out kinases, we also used the program PASS [31] to investigate the potential binding sites in the active-site cleft. The PASS results showed that there exist three binding pockets in the active-site clefts of the DFG-out structures. For example, in Fig. 1D the centers of the three pockets in the ABL1 DFG-out structure (PDB code: 2F4J) are shown as red spheres, B_1_, B_2_ and B_3_, respectively. These three pockets were also defined as adenine pocket, hydrophobic pocket II and DFG-out pocket, respectively, in the study by Ranjitkar et al. [18] (see figure 1B in this reference). To quantitatively define these three pockets, we also used the program LIGSITE [32] to calculate the numbers of 1 Å-grid points in the pockets. The numbers of the LIGSITE grid points in the pockets are related to their volumes. For the sake of intuition, we simply transformed the numbers of the grid points into the numbers of water molecules in a density of 1 g⋅ml^−1^: any water molecule was considered as non-occupied if its nearest distance to the grid points of the three pockets is large than 1.6 Å. The numbers of the occupied water molecules in the mentioned pockets for some known DFG-out structures were listed in Table 3. Note that, some crystal DFG-out structures in Table 1 lacked certain atomic coordinates in their activation-loops, and thus their data of occupied water molecules are not presented in Table 3. Again, the numbers of occupied water molecules in Table 3 indicate that a wide active-site cleft is crucial for the specific binding of type-II inhibitors to the DFG-out conformation of a protein kinase. In other words, a type-II inhibitor which is able to simultaneously bind to the three pockets in the DFG-out conformation should possess a scaffold of certain volume and length, as indicated by Ranjitkar et al. [18].

**Table 3:** Numbers of occupied water molecules in the active-site clefts of kinase DFG-out structures in complex with type-II inhibitors.

Kinases	PDB codes	Numbers of occupied water molecules
ABL1	1FPU	46
	1IEP	49
	1OPJ	50
	2HIW	41
	2HYY	43
	2HZ0	33
	2HZN	45
KIT	1T46	43
LCK	2PL0	45
MK14	1W82	44
	1W83	35
	1WBN	45
	1WBS	44
	1WBT	42
	1WBV	39
	2BAJ	44

### Predictions of kinase DFG-out models

**Figure 2:**
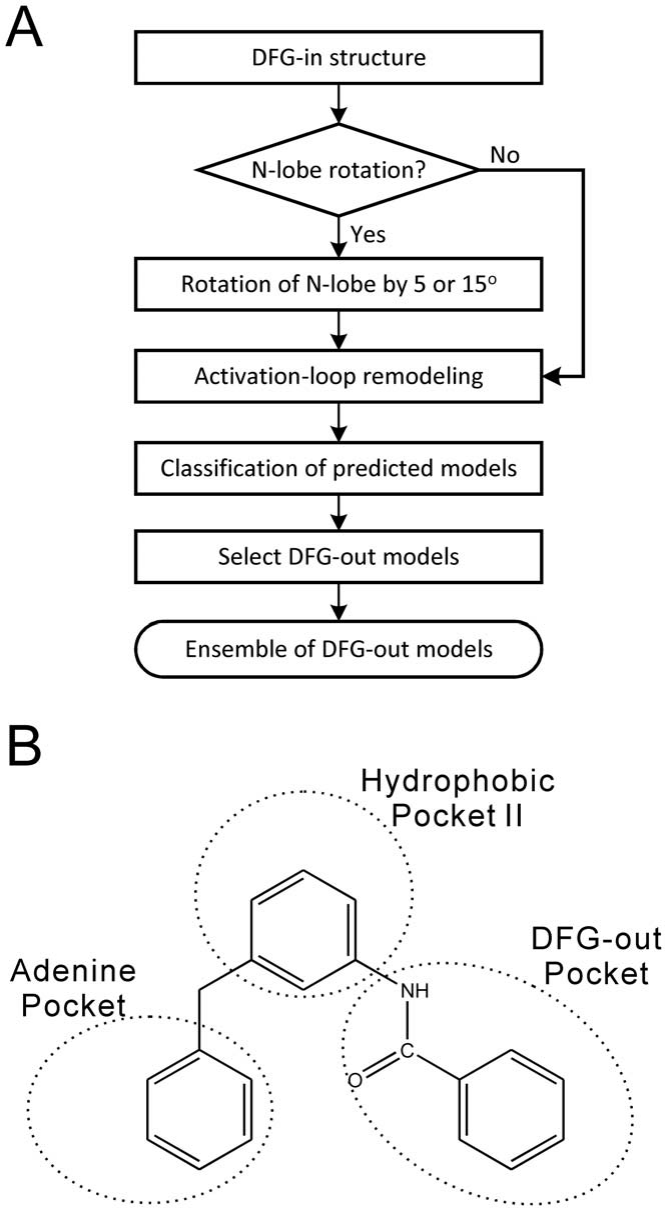
The activation-loop remodeling method (ALRM) and the vitual molecule defined for selecting the DFG-out models. (A) Flowchart of the ALRM approach. (B) The structure of the virtual molecule that resembles the minimum core scaffold of typical type-II inhibitors.

For each protein kinase listed in Table 1, we employed the ALRM approach illustrated in Fig. 2A to generate 200 lowest-energy models using its corresponding DFG-in structure, and then classified them into DFG-in or DFG-out models according to the method described in Materials and Methods. As indicated in Table 4, about 31–55% of the lowest-energy models were found to be in the DFG-out conformations. Like other proteins, kinases are dynamic macromolecules which are able to explore multiple conformations via internal motions. As pointed out by Aleksandrov and Simonson [33], according to equilibrium statistical mechanics even the highest energy conformations of an apo-kinase have non-zero populations. Thus, the high percentages of the lowest-energy models with the DFG-out conformation imply that certain low-energy DFG-out conformations might be sampled dynamically by the kinase. In fact, the crystal structures of kinases in complex with type-II inhibitors have already implied that multiple DFG-out conformations do exist at physiological conditions and may be trapped and stabilized by the inhibitors [34]. Of course, whether the DFG-out conformations could stay stable or just transiently exist remains an open question, and further investigations by other approaches beyond the scope of this study are needed, such as by molecular dynamics (MD) simulation.

**Table 4:** The percentages of DFG-out models, populations of major activation-loop clusters, TM-score values and heavy-atom RMSDs of DFG-motif with respect to the corresponding crystal structures.

Kinases	PDB codes of DFG-out structures	Structural resolution (Å)	Numbers and percentages of DFG-out models	Populations of DFG-out clusters ≥5	Global TM-score		DFG motif RMSD (Å)	
					Average	Maximum	Average	Minimum
ABL1	1FPU	2.40	104 (52%)	52, 20, 10, 6	0.86	0.89	4.48	1.64
BRAF1	1UWH	2.95	67 (34%)	41, 13, 5, 5	0.87	0.88	4.45	1.45
EPHA3	3DZQ	1.75	101 (51%)	68, 17, 7	0.90	0.91	5.71	1.51
KIT	1T46	1.60	67 (34%)	39, 12, 6, 5	0.88	0.90	5.39	2.03
LCK	2PL0	2.80	102 (51%)	56, 21, 7, 6	0.89	0.92	4.59	1.98
MK14	1WBT	2.00	61 (31%)	41, 9	0.95	0.97	5.02	2.03
SRC	2OIQ	2.07	109 (55%)	56, 23, 11, 7	0.96	0.97	2.75	0.60

It is well known that the activation-loops of PKs are flexible segments. Indeed, this flexibility is also the reason why in a lot of crystal structures of PKs the atomic coordinates of the part or whole activation-loop were unable to be solved. Also, flexibility means that the activation-loops may explore multiple conformations in a dynamic way and possess certain conformational diversity, for example, the activation-loop of ABL1 in the DFG-out conformation has been observed to adopt two very different conformations (see details in PDB codes: 1FPU and 2HZ0). To characterize such conformational diversity, we performed clustering analysis using the MaxCluster program (see http://www.sbg.bio.ic.ac.uk/~maxcluster/index.html) based on the activation-loop conformations in the DFG-out models. We used MaxCluster because it was able to effectively deal with the conformational comparison of short segments among proteins, e.g., the activation-loops. To carry out the conformational comparison required for clustering, we considered a segment of sixteen residues which starts from the second preceding residue of the DFG motif and covers the whole activation-loop in the studied kinases. Based on the conformations of this segment in the DFG-out models, the predicted DFG-out models of the studied kinases were then clustered using the nearest neighbor clustering algorithm with an RMSD (root mean square deviation) threshold of 4 Å, and the populations of the major conformational clusters with members not fewer than 5 are listed in Table 4.

**Figure 3:**
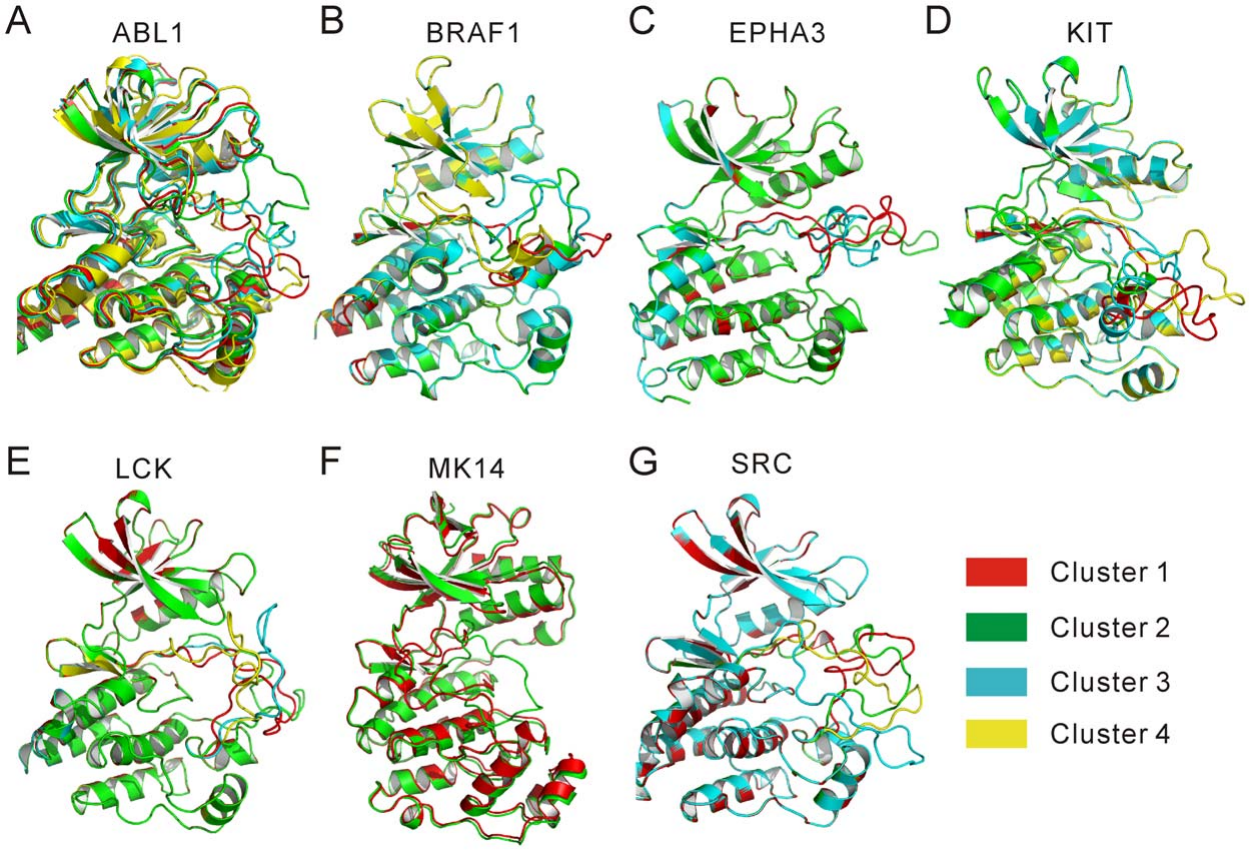
Representative DFG-out models of the major activation-loop clusters. (A) ABL1. (B) BRAF1. (C) EPHA3. (D) KIT. (E) LCK. (F) MK14. (G) SRC.

As seen in Table 4, for all 7 kinases the numbers of major DFG-out activation-loop clusters are about 2–4. This implies that the activation-loops of these kinases possess certain flexibility, in agreement with the experimental observation that one kinase can access multiple inactive conformations [10], [34]. To demonstrate this conformational diversity, the representative conformations of the major clusters of each kinase in Table 4 are illustrated in Fig. 3. As shown, the activation-loops of ABL1, BRAF1, KIT, LCK and SRC may adopt more different conformations, while those of EPHA3 and MK14 adopt fewer. The conformational diversity of the activation-loops may be attributed to the central and C-terminal parts of the loops, and the N-terminal parts are relatively stable. This is reasonable because the central and C-terminal parts of the activation-loops are more exposed to the solvent. The cluster populations in Table 4 also reveal an interesting fact that there exists a large cluster (i.e., the first cluster) in each kinase which contains more than 50% of the DFG-out models, and thus the populations of the other clusters are relatively small. Since the DFG-out models were obtained by independent Rosetta runs, the large population of the first cluster might imply that the conformations in this cluster are the most probable DFG-out conformations of the kinase. Of course, due to the transient nature of the DFG-out conformations, many of them may not be observed directly, and those in the crystal structures appear to be a few probable DFG-out conformations which were trapped and stabilized by the type-II inhibitors.

To examine the relationship between the predicted DFG-out models and the corresponding crystal DFG-out structures, we also carried out structural comparison in two different ways: global comparison which focuses on the whole structure using the TM-align program [35], and local comparison which focuses on the DFG motif using the MaxCluster program. Results of the TM scores and the heavy-atom RMSDs of the DFG motifs are listed in Table 4. Results in Table 4 show that TM-scores are all greater than 0.85, indicating that the predicted DFG-out models as a whole are very similar to the crystal structures in complex with the type-II inhibitors. This is attributed to that, with respect to the crystal structures, in the DFG-out models no significant conformational change occurs in the C-lobes, and the N-lobes essentially maintain the scaffolds similar to those in the crystal structures.

**Figure 4:**
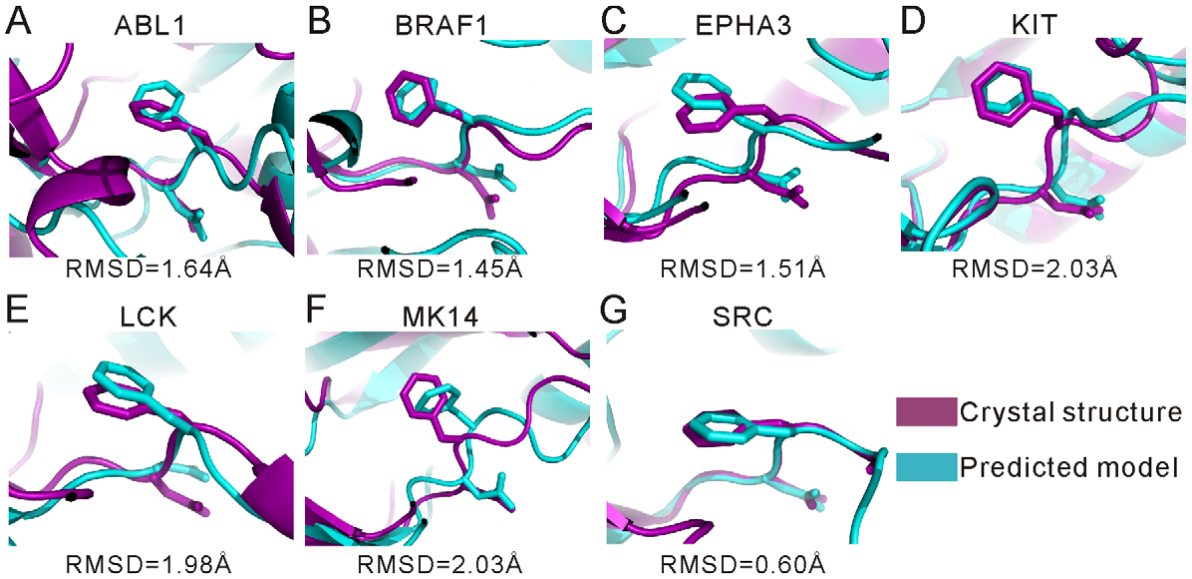
Superposition of DFG motifs in the DFG-out models with respect to those in the crystal structures. (A) ABL1. (B) BRAF1. (C) EPHA3. (D) KIT. (E) LCK. (F) MK14. (G) SRC.

On the other hand, as seen, for the local comparison of the DFG motifs the average RMSDs are in the range from 4.0 to 6.0 Å. Again, this indicates the conformational diversity of the activation-loops, and demonstrates that the ALRM approach could sample multiple DFG-out conformations without bound type-II inhibitors. Despite of the conformational diversity, the minimum RMSDs were found to be below or around 2.0 Å, and the superposition of the predicted DFG motifs with the minimum RMSDs against the crystal structures indicated that the predicted models are in good agreement with the crystal structures (Fig. 4). This suggests that the DFG-out conformations close to those in the crystal structures could be sampled by the ALRM approach. Because the conformation of the DFG motif is the major site targeted by the type-II inhibitors, and also vital for the formation of a wide active-site cleft, the ability of the ALRM approach to generate conformations of which DFG motifs are similar to those in the crystal structures shows its potential for design and discovery of the type-II inhibitors.

Thus, our ALRM approach based on the DFG-in structures could predict multiple DFG-out conformations of protein kinases reliably. Compared with the DOLPHIN model [28], our method deletes no residues and the DFG-out models possess all atomic coordinates of the activation-loop residues. Also, as indicated by Table 4, our approach is able to sample possible DFG-in and DFG-out conformations of a kinase, and therefore mimics the dynamic conformational ensembles of the kinase. The ability of the current approach to sample a large number of possible activation-loop conformations ensured us to generate certain DFG-out conformations which may be targeted by specific type-II inhibitors. Recently, it has been shown that the DFG-flip from the DFG-in to DFG-out conformations could also be triggered through MD simulation [11]. However, because the flip appears to have to overcome a high energy barrier, to simulate the flip process at the atomic level needs long time-scale calculations. Compared to the MD simulation, the current approach is faster when sampling a large number of possible conformations. And this is also important for the practical applications in the structure-based drug design and discovery.

Because in the ALRM approach the numbers of the obtained DFG-out models are in the range of tens to hundreds, in practice it becomes important to select a small number of models for molecular docking in order to reduce computational cost. Of course, in principle one could use all predicted DFG-out structures in the binding pose prediction and virtual screening. However, as mentioned, known type-II inhibitors were found to occupy three potential pockets (i.e., adenine pocket, hydrophobic pocket II and DFG-out pocket) in the DFG-out conformations and thus possess relatively large scaffolds. For example, the molecular weights of the known type-II inhibitors in Table S1 of Supporting Information are in the range from 287 to 594. It is obvious that DFG-out structures with small active-site clefts could not accommodate large type-II molecules. To avoid unnecessary docking to the DFG-out structures with small active-site clefts, in this study we only selected the DFG-out structures with large active-site clefts.

As discussed in the last subsection, typical type-II inhibitors possess a scaffold of certain volume and length in order to simultaneously bind to the three pockets of the DFG-out conformation as shown in Fig. 1D. Thus, inspired by the study of Ranjitkar et al. [18], we derived a virtual molecule which roughly resembles the minimum core scaffold of typical type-II inhibitors (Fig. 2B). By using the same way to calculate the volume of active-site cleft as described in the next subsection, we found that this virtual molecule occupies a space of about 20 water molecules. Thus, after several preliminary tests, we eventually used the criterion of 20 occupied water molecules for DFG-out model selection: only those models satisfying the selection criterion were chosen to form a DFG-out conformation ensemble. On average, the numbers of the selected DFG-out models for the kinases in Table 1 are about 10, and therefore the computational costs are significantly reduced as compared to the use of all the predicted DFG-out conformations. Note that, in the following virtual screening the use of DFG-out structures with large active-site clefts does not eliminate small-sized compounds in consideration. In fact, both small-sized and large-sized compounds were fully considered when using the DFG-out structures with large clefts, because these structures could accommodate not only large-sized compounds, but also small-sized compounds whose volume is smaller than 20 occupied water molecules.

### Binding pose predictions of the known type-II inhibitors

As mentioned, a potential application of the predicted DFG-out models in the structure-based drug design is to predict the binding modes of type-II inhibitors to protein kinases. To explore such a possibility, we performed molecular docking with the known type-II inhibitors using the selected DFG-out models as the receptors. Molecular docking with the program AutoDock (Version 4.2) [36], [37] was carried out for the inhibitor-kinase complexes listed in Table 5. The molecular structures of these type-II inhibitors are listed in Table S1 of Supporting Information.

**Table 5:** RMSDs of the lowest-energy representative poses of type-II inhibitors with respect to those in the crystal structures using DFG-out models and crystal structures, respectively.

Kinases	Type-II ligands	Based on DFG-out models		Based on crystal structures
		RMSD of the lowest-energy pose (Å)	Minimum RMSD (Å)	RMSD of the lowest-energy pose (Å)
ABL1	406	1.73	1.30	1.13
	7MP	2.05	0.75	0.96
	GIN	2.59	1.21	0.84
	KIN	1.49	0.71	9.91^a^
				1.47^b^
	PRC	9.57^a^	0.85	2.08
		1.48^b^		
	STI	2.78	2.51	0.54
BRAF1	BAX	1.77	1.77	1.04
EPHA3	IFC	1.88	1.69	1.05
KIT	STI	1.94	1.76	0.99
LCK	1N8	2.00	1.95	1.11
	242	1.97	1.68	0.99
	9NH	2.27	2.16	6.40
	STI	2.56	2.21	1.16
MK14	1PP	1.42	1.42	1.50
	AQZ	1.93	1.93	2.36
	B96	2.17	2.17	1.15
	BMU	1.34	1.34	0.87
	L09	1.69	1.69	1.67
	L10	1.47	1.47	0.66
	L11	1.63	1.54	1.73
	LI2	1.34	1.13	1.53
	LI3	0.68	0.62	1.29
	WBT	1.47	1.44	1.61
SRC	STI	1.94	1.88	1.28

a The lowest-energy poses possess an opposite orientation with respect to those in the crystal structures, and therefore have large RMSDs.

b The minimum RMSDs of binding poses other than the lowest-energy poses.

For each inhibitor, molecular docking to all selected models in the DFG-out ensemble of the given kinase was first conducted with AutoDock, and then the predicted binding poses were extracted and analyzed by this procedure: the docking poses outside the active-site clefts of all selected DFG-out models were ruled out at first; then, the remaining poses in all selected DFG-out models were ranked according to their docking energies; finally, the lowest-energy pose was treated as the representative pose of this inhibitor, and the corresponding DFG-out model bound by the representative pose of the inhibitor was regarded as the representative DFG-out conformation of the kinase. Consequently, the representative pose of the ligand for each inhibitor-kinase complex was compared to that in the corresponding crystal structure, and the results of the heavy-atom RMSDs are listed in Table 5.

**Figure 5:**
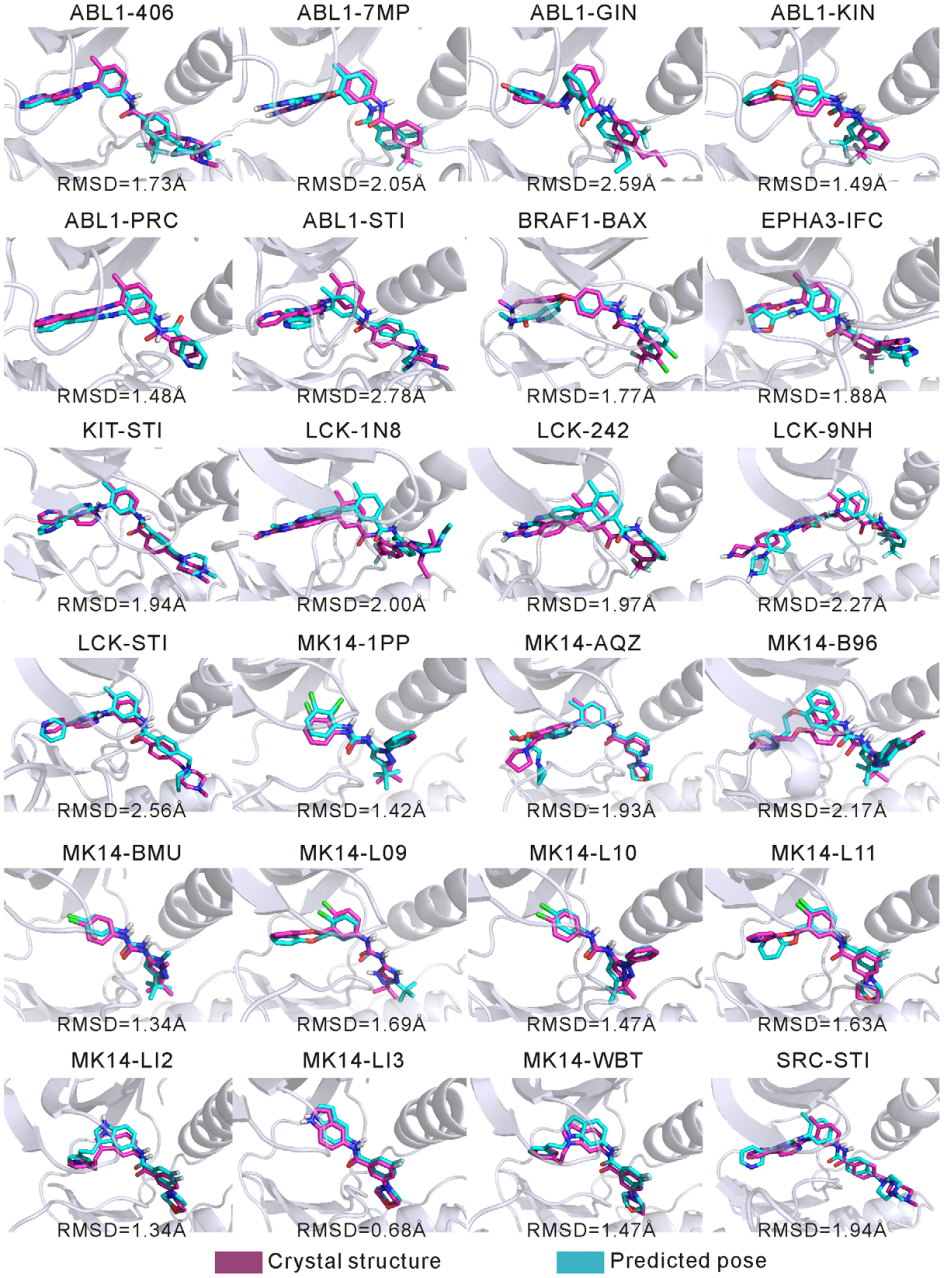
Superposition of the predicted binding poses of type-II ligands with respect to those in crystal structures for 24 known kinase-inhibitor pairs.

Table 5 shows that the RMSD values of the lowest-energy poses with respect to those in the crystal structures are below or around 2 Å, the usual RMSD criterion for determining the quality of predicted poses with respect to the crystal structures. By a careful analysis of all the docking poses, we found that for almost all inhibitors the binding poses could be classified roughly into 2 major clusters: poses in one cluster are similar to that in the corresponding crystal structure and thus possess small RMSD values, and poses in the other cluster have an opposite orientation to that in the crystal structure and therefore possess large RMSD values. As indicated by RMSDs in Table 5, except in one kinase-ligand pair (i.e., ABL1-PRC), the predicted lowest-energy poses of the ligands were found to have the same orientations as those in the crystal structures. Thus, the predicted orientations of the type-II inhibitors were in very good agreement with those in the crystal structures, with a success rate close to 96%. This can also be seen in the superposition of the lowest-energy poses of type-II ligands with their crystal structures (Fig. 5). Since in the molecular docking simulations the protein flexibility for a given DFG-out model was not considered, this could cause errors in the binding pose predictions. For example, for several kinase-ligand pairs, although the orientations of the lowest-energy poses of the ligands were the same as those in the crystal structures, their RMSD values are greater than 2.0 Å. Thus, we further examined the RMSDs of the docking poses possessing similar orientations as those in the crystal structures. Results for the poses with the minimum RMSDs are given in Table 5. As seen, about 83% of the minimum RMSDs are less than 2.0 Å. Again, this indicates that the molecular docking approach in this study could reliably predict the binding poses of type-II inhibitors to protein kinases.

To further examine the reliability of the docking procedure using the DFG-out models, we also perform docking using the crystal structures listed in Table 1 as the receptors, with the same protocol as described in above. The RMSDs of the lowest-energy binding poses are given in Table 5. Except for ABL1-KIN and LCK-9NH, most of the lowest-energy poses are in good agreement with those in the crystal structures. Especially, the RMSD values of twenty lowest-energy poses are smaller than 2.0 Å. This number is the same as that of the minimum RMSDs using the DFG-out models. These results not only demonstrate that the employed docking procedure based on AutoDock 4.2 is reliable, but also suggest that the DFG-out models are valid for type-II inhibitor docking.

**Figure 6:**
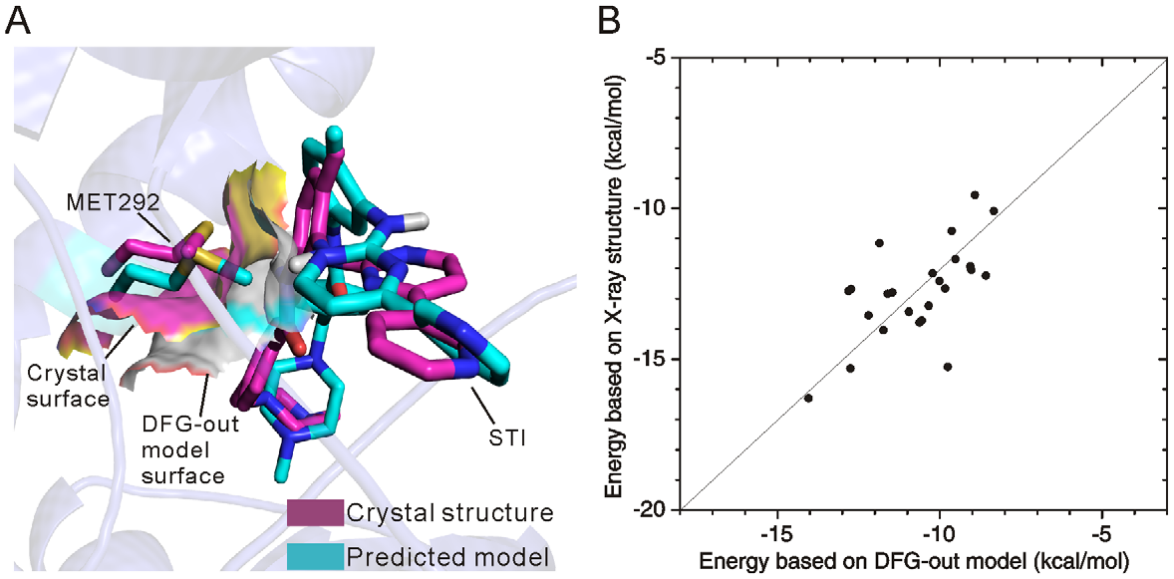
Docking pose with a relatively large RMSD and correlation of docking energies. (A) Steric clash in the DFG-out models of LCK. (B) Correlation of the docking energies based on the DFG-out models with those based on the crystal structures.

As mentioned, the RMSDs of several lowest-energy poses in Table 5 are greater than 2.0 Å. For example, for kinase LCK even the minimum RMSDs are close to or greater than 2.0 Å. Comparing the bound DFG-out models with the corresponding crystal structure, we found that the relatively large RMSDs were mainly caused by different conformations of residue Met292 in the DFG-out models and the crystal structure (PDB code: 3LCK). In the DFG-out models, the side-chain of Met292 usually extends towards the hydrophobic pocket II and thus hinders the binding of the ligands to the optimal position. In contrast, in the crystal structure, the side-chain of Met292 adopts a different orientation to leave enough space for the inhibitor binding (Fig. 6A). Besides, other factors may also affect the binding poses, such as the conformation of the Lys273-Glu288 salt bridge etc. The residue Glu288 usually forms two hydrogen bonds with the Lys273 and the type-II inhibitors, respectively, to stabilize the binding poses. So either the steric effect or the missing hydrogen bonds may affect accuracy of the binding pose prediction. To overcome such a problem, in the future it seems we need a method combining the current approach with MD simulation in order to account for additional small-scale motions in the kinase-inhibitor complex obtained by AutoDock, like the MD refinements of the docked HIV-1 protease-inhibitor complexes [38], [39].

Since the crystal structures are the DFG-out conformations bound by the type-II inhibitors and the key residues in the active-site clefts affecting the binding pose are in their optimal positions, the docking energies of the lowest-energy poses obtained by these structures could be used as a reference to validate the AutoDock docking energies based on the DFG-out models. The correlation between the docking energies based on DFG-out models and the crystal structures is illustrated in Fig. 6B. As seen, most of the docking energies of the lowest-energy poses based on the DFG-out models correlate well with those based on the crystal structures. In Fig. 6B, there exists a systematic shift of ∼2.0 kcal/mol between the docking energies based on the DFG-out models and those based on the crystal structures. Such an energy shift might be attributed to that not all the residues of the DFG-out models are in their optimal positions for the binding of the inhibitors, because the DFG-models were generated with the ALRM approach without any bound inhibitors, and in the docking process, the DFG-out models were set as rigid receptors. Recently, studies have shown that there exist certain differences between the predicted binding free energies and the observed energies of the type-II inhibitors [28], [33], and this is mainly because in molecular docking many factors affecting the observed binding free energies were neglected, such as population differences between the DFG-out and DFG-in conformations in solution [18]. Nevertheless, to a certain extent the good correlation between the docking energies using the DFG-out models and those based on the experimental structures indicates that the docking procedure itself is reliable, and the AutoDock score could be used to rank the docking poses based on the DFG-out models. Of course, further analysis is needed to find out the exact differences between the AutoDock energies and the observed energies.

### Virtual screening of type-II inhibitors using the DFG-out models

To test whether the predicted DFG-out models are applicable to virtual screening of the type-II inhibitors, we carried out a virtual screening study using the ensembles of DFG-out models and a database of about 750 known protein inhibitors commercially available from MERCK (i.e., Calbiochem inhibitor database). The Calbiochem inhibitor database is a collection of various potein inhibitors, and its details can be seen in *Inhibitor SourceBook* (2rd Edition) by MERCK. The inhibitor files suitable for molecular docking were downloaded from a homepage of ZINC website [40] at http://zinc.docking.org/vendor0/index_fsm.shtml (Please see the item related to Calbiochem on this page). To test whether the DFG-out models can recognize their specific type-II ligands, the inhibitors listed in Table S1 were also included into the screening database. Because the molecular size in the screening database may have effects on the study, we calculated the molecular weights of the database inhibitors and compared them with those of the known type-II inhibitors. The molecular weights of the inhibitors in the database are in the range from 100 to 550, and the distribution is shown in Figure S1 in Supporting Information. Meanwhile, the molecular weights of the known type-II inhibitors in Table S1 of Supporting Information are in the range from 280 to 594. Thus, there is no significant difference in the molecular size between the dataset and the known type-II inhibitors.

**Figure 7:**
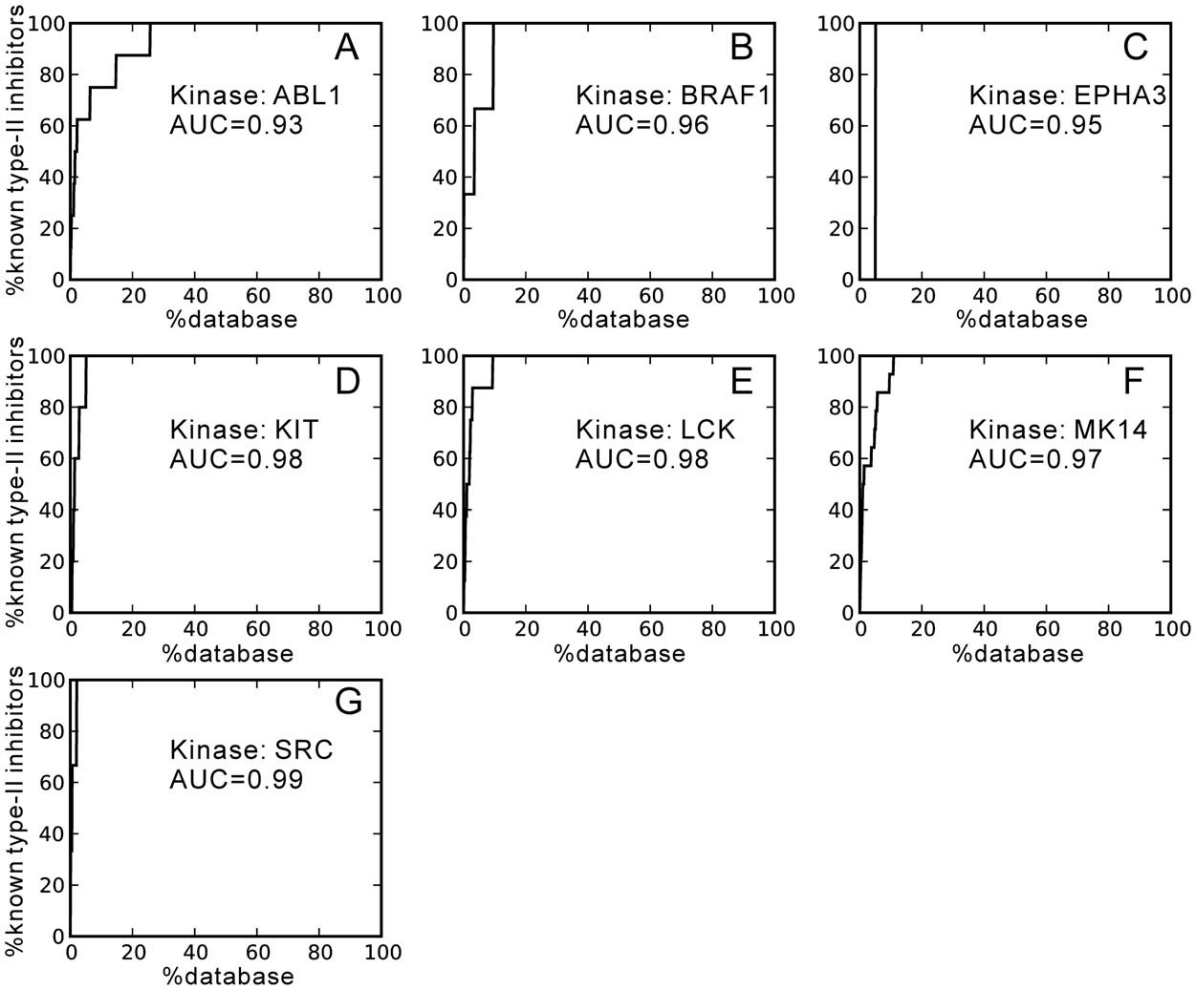
Performance of virtual screening for type-II inhibitors using the predicted DFG-out models, as shown by the AUC (area under curve) values. (A) ABL1. (B) BRAF1. (C) EPHA3. (D) KIT. (E) LCK. (F) MK14. (G) SRC.

In the virtual screening for a given kinase target, molecular docking was performed for each inhibitor in the database against every model in the DFG-out ensemble of the given kinase. The representative for a given inhibitor was selected by the same procedure as described in the binding pose predictions of known type-II inhibitors: the lowest-energy pose of the ligand in all DFG-out models was used as the binding representative of the inhibitor to the kinase target. Eventually, all inhibitors for the given kinase target were re-ranked by the docking energies of their representatives to form a hit list from the lowest-energy inhibitor to the highest-energy inhibitor. Based on the obtained hit lists, AUC (area under ROC curve) values which characterize the virtual screening performance were calculated, as shown in Fig. 7.

As seen in Figs. 7A–G, all the AUC values of the target kinases are greater than 0.90. Such high AUC values indicate that the predicted DFG-out models are selective toward their specific type-II inhibitors. Indeed, all the known type-II inhibitors were found to be ranked in the top 1–5% in their hit lists. More importantly, consistent with the results of binding pose prediction in Table 5, the binding poses of the top-ranked hits were also similar to those in the crystal structures. Therefore, these results illustrate that the computational approach and protocols presented in this study are very promising for structure-based screening of novel type-II inhibitors of protein kinases.

As mentioned, in the past there were few effective methods able to identify the type-II inhibitor targeting a specific DFG-out conformation for a given protein kinase. To address this issue, here we employed a conformational selection procedure that used an ensemble of DFG-out conformations in the binding pose predictions and virtual screening. In this approach, as the representative binding pose of the ligand was identified, the DFG-out conformation in complex with the representative pose was determined, too. The results of the binding pose predictions and virtual screening demonstrate that the conformational selection protocols developed in the study are effective for the identification of the DFG-out conformation targeted by specific inhibitors. From the viewpoint of dynamic conformational ensembles of proteins, we may consider this procedure as the process that the given type-II ligand selects its most favorite DFG-out conformation [41]. If one postulates that the DFG-in and DFG-out conformations of a protein kinase are populated (i.e., pre-existing) in solution, in general, different ligands may bind to and stabilize different populated DFG-in and DFG-out conformations, e.g., different ABL1 inhibitors [25]. So there is no doubt that the conformational selection procedure using a representative ensemble of DFG-out conformations would be a more effective way to discover specific kinase inhibitors than using only one DFG-out conformation. Thus, computational approaches for reliably predicting kinase DFG-out structures are valuable for the structure-based drug design and discovery of protein kinase inhibitors.

### Conclusion

In this study, we have developed a computational approach for predicting the inactive DFG-out conformations of protein kinases using the existing DFG-in structures, and developed conformational selection protocols for the applications of the predicted DFG-out models in the binding pose prediction and virtual screening of the type-II inhibitors. Using the DFG-out models, we predicted the binding poses for the known type-II inhibitors, and the results were found to be in good agreement with the X-ray crystal structures. Also, we tested the abilities of the DFG-out models to recognize their corresponding type-II inhibitors by screening a database of small molecules. The AUC results indicated that the predicted DFG-out models are selective toward their specific type-II inhibitors. Therefore, these results illustrate clearly that the computational approach and protocols presented in this study are very promising for the structure-based design and screening of novel type-II inhibitors targeting protein kinases.

## Methods

### Preparation of the DFG-in structures

The DFG-in structures for the DFG-out conformation predictions were downloaded from the Protein Data Bank. For each kinase structure, ions and small molecules including water were first deleted. Then, if the kinase possesses phosphorylated groups, those groups were mutated into the original non-phosphorylated amino-acids using the homology-modeling program MODELLER [42], and if there were missing atoms in the PDB files, the coordinates of the missing atoms were also added by standard modeling procedure with MODELLER. Then, the DFG-in structures were refined by using the high-resolution protocols implemented in the Rosetta program [29]. Finally, the distance sum of the four conserved residues (LCK numbering: Lys273, Glu288, Asp and Phe of the DFG-motif, see PDB code: 3LCK) was calculated, and the N-lobe of the kinase domain was rotated according to the degree values defined in Table 2.

### Activation-loop remodeling

To predict the DFG-out conformation, activation-loop remodeling of the DFG-in structure was performed using the rebuilding-and-refinement protocols implemented in the ‘loop_relax’ subroutine of the Rosetta program [43]. Fragment files, loop definition file and other parameters needed for running the Rosetta program were created and defined according to the protocols. The loop segment for the modeling was defined to start with the second N-terminal residue ahead of the DFG motif, and end at the last C-terminal residue of the activation-loop. We used the most hydrophilic residue located in the middle part of the defined loop segment as the cut point. The cyclic coordinate descent (CCD) method was used to maintain the chain connectivity of the loop segment in the modeling process [44]. After the loop segment rebuilding, all-atom refinement procedure was employed to refine the side chains of the activation-loop rebuilding model and thereby obtain the final all-atom lowest energy conformation. For each kinase, 200 all-atom lowest energy models were generated by independent runs. The whole procedure for predicting one all-atom model took approximately 3–4 CPU hours on a usual Intel Pentium IV processor. And all the model computations were conducted on the MagicCube supercomputer in Shanghai Supercomputer Center, China.

### Conformational classification of predicted models

**Figure 8:**
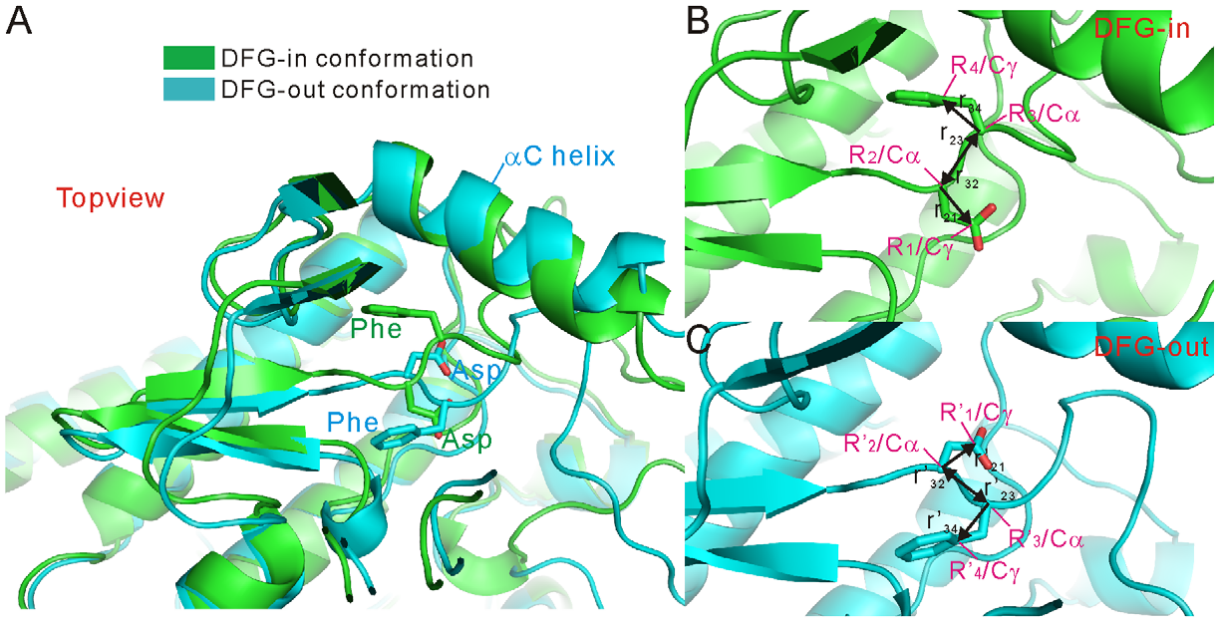
Vectors defined for conformational classification of predicted kinase models. (A) Topview of the aligned DFG-in and DFG-out structures. (B) Vector definitions in the DFG-in structures. (C) Vector definitions in the DFG-out structures.

The obtained models were first classified into DFG-in, DFG-out and intermediate conformations by the following vector-based method. This method is based on that before and after the DFG-flip process the Asp and Phe residues are always located on two opposite sides of the activation-loop (Fig. 8A). Thus, by aligning the obtained model against the starting DFG-in structure, if the Asp and Phe residues of the model is on the same sides of the corresponding residues of the DFG-in structure, that model would be considered as a DFG-in model; if the situation is just opposite, that model would be classified into the class of the DFG-out conformations. All other models are classified as the intermediate conformations. To the end, four atoms of the DFG-motif were selected from each aligned structure, C_γ_ and C_α_ atoms of Asp, C_α_ and C_γ_ atoms of Phe, and labelled as 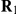, 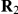, 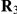 and 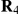 for the atoms of the starting DFG-in structure, and 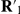, 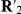, 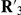 and 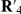 of the obtained model, respectively. Then, eight vectors are defined as (Figs. 8B and C):
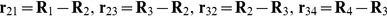
(1)

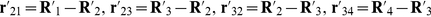
(2)By cross products of these vectors, four new vectors are derived as:
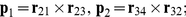
(3)

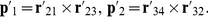
(4)If the directions of the vectors 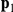 and 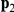 are opposite, the point multiplication of the two vectors is negative. Therefore, after aligning a predicted model against the starting DFG-in structure, if 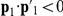 and 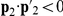, this model is a DFG-out model; if 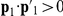 and 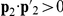, this model is considered as a DFG-in model. Other models are treated as models in the intermediate state of the DFG-flip.

### Selection of DFG-out models for molecular docking

To select appropriate DFG-out models for molecular docking, the classified DFG-out models were analyzed by the programs PASS [31] and LIGSITE [32], in order to identify the putative binding sites in the active-site cleft, and characterize the volumes of the binding pockets. To ensure that the active-site cleft is large enough for accommodating a type-II inhibitor, only those DFG-out models with at least three binding sites identified by PASS in the active-site cleft were first selected. These selected models were then analyzed with the program LIGSITE to find out the actual volumes of their active-site clefts. LIGSITE determined the pocket space by calculating the numbers of 1 Å-grid points in the active-site cleft, and the number of the grid points is related to the pocket volume. For the sake of intuition, the number of the grid points was transformed into the number of water molecules in the density of 1 g⋅ml^−1^ in the cleft: any water molecule was considered as non-occupied as its nearest distance to the grid points of the three pockets was greater than 1.6 Å. To ensure the finally selected models possessing an active-site cleft with certain space, we employed a number of occupied water molecules more than 20 as the criterion to select the final DFG-out models to form the ensemble of DFG-out conformations for molecular docking.

### Ensemble docking of the DFG-out models

AutoDock program (Version 4.2) with the Lamarckian genetic algorithm [36], [37] was used for the molecular docking in the binding pose prediction and virtual screening. We used the central binding site B_2_ identified by the program PASS as the center of the grid box for docking, see Fig. 1D. And the size of the grid box is 60×60×60 Å. Twenty docking runs of a ligand were carried out for each DFG-out conformation of the target kinase. To conduct the Lamarckian genetic algorithm, a population of 150 random ligand conformations in random orientations and at random translations was first generated, and then the population evolved according to the algorithm and terminated after 27,000 generations and a maximum of 1,500,000 energy evaluations. Eventually, for a given pair of ligand and the DFG-out conformation, the docking yielded 20 docked poses. Other necessary parameters for docking were set to the default values provided by AutoDock. After the docking for all DFG-out models of the ensemble was completed, all the docking poses for the given kinase-ligand pair were collected and analyzed by the following protocol of docking pose analysis.

### Analysis of docking poses

The docking poses of a ligand against all DFG-out models in the ensemble of a target kinase were extracted and analyzed. Any docking pose in which no atoms whose smallest distance to the PASS-defined binding site points B_2_, B_3_ (see Fig. 1D) was less than 3 Å was considered as outside the active-site cleft and ruled out at first. The remaining poses from all DFG-out models were then ranked according to their docking energies. The lowest-energy pose was treated as the representative pose of the ligand, and the corresponding DFG-out model in complex with the ligand representative was considered as the representative DFG-out model.

## Supporting Information

Figure S1 Distribution of molecular weights of the inhibitors in Calbiochem inhibitor database.
(DOC)

Table S1 Molecular structures of the type-II inhibitors used in the study and the names of their target kinases.
(DOC)
