# Supplementary material for: Predicting Inactive Conformations of Protein Kinases Using Active Structures: Conformational Selection of Type-II Inhibitors
Source: PLoS One. 2011 Jul 27;6(7):e22644. doi: 10.1371/journal.pone.0022644 (PMC3144914; doi:10.1371/journal.pone.0022644)

**Supporting Information**

Figure S1. Distribution of molecular weights of the inhibitors in Calbiochem inhibitor database.


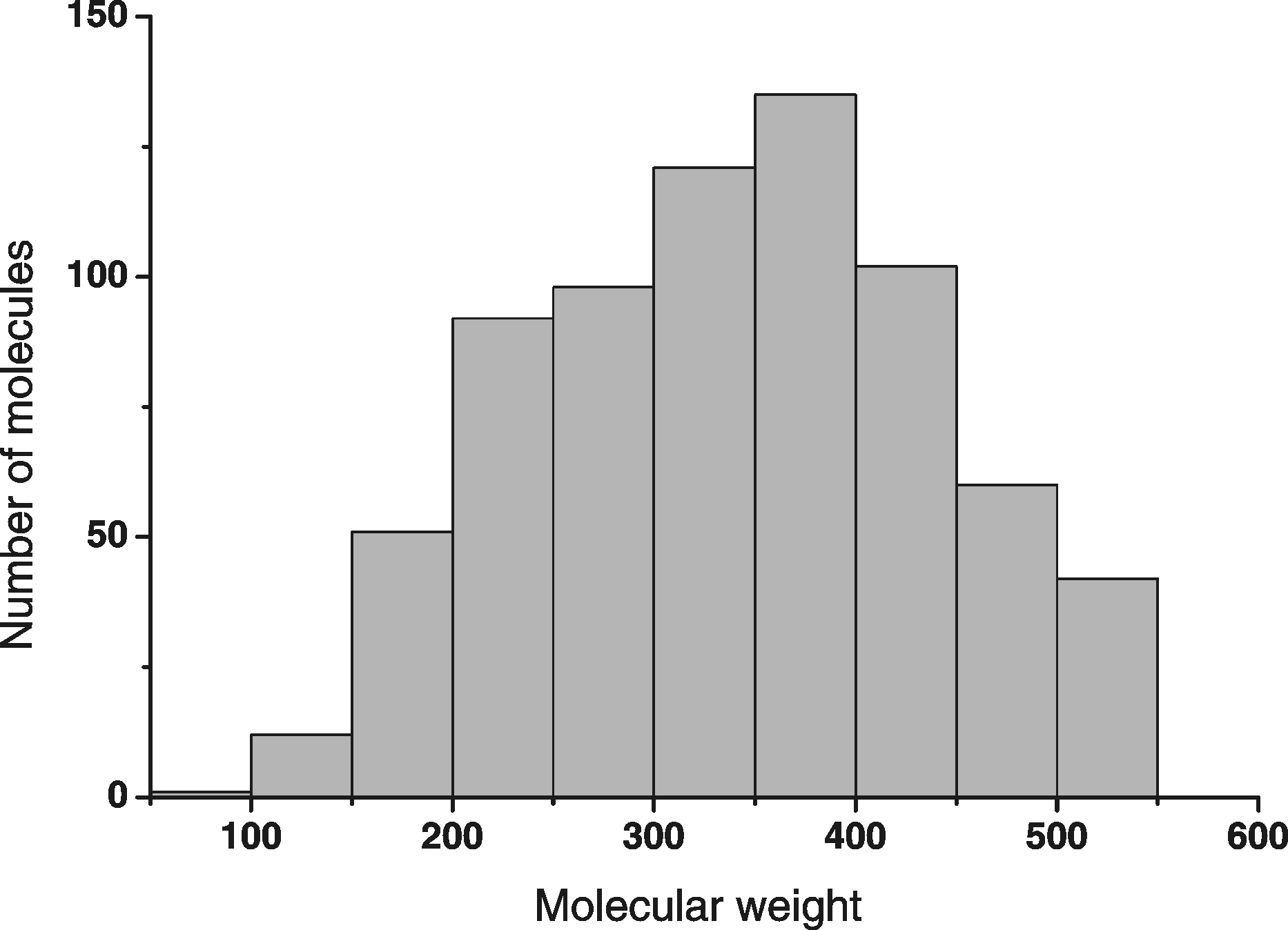

Supplement: Figure S1 — Distribution of molecular weights of the inhibitors in Calbiochem inhibitor database. (DOC) [file pone.0022644.s001.doc]
