# Supplementary material for: Predicting Inactive Conformations of Protein Kinases Using Active Structures: Conformational Selection of Type-II Inhibitors
Source: PLoS One. 2011 Jul 27;6(7):e22644. doi: 10.1371/journal.pone.0022644 (PMC3144914; doi:10.1371/journal.pone.0022644)
Supplement: Table S1 — Molecular structures of the type-II inhibitors used in the study and the names of their target kinases. (DOC) [file pone.0022644.s002.doc]

**Supporting Information**

Table S1. Molecular structures of the type-II inhibitors used in the study and their target kinase names.

| 1N8 (LCK, MK14) | 1PP (MK14) | 242 (LCK, MK14) |
| --- | --- | --- |
| 406 (ABL1) | 7MP (ABL1, KIT, LCK, SRC) | 857 (KIT, LCK, SRC) |
| 9NH (LCK, MK14) | AQZ (MK14) | B96 (ABL1, BRAF1, KIT, LCK, MK14) |
| BAX (ABL1, BRAF1, KIT, LCK, MK14) | BMU (MK14) | GIG (BRAF1) |
| GIN (ABL1) | KIN (ABL1) | IFC (EPHA3) |
| L09 (MK14) | L10 (MK14) | L11 (MK14) |
| LI2 (MK14) | LI3 (MK14) | PRC (ABL1) |
| STI (ABL1, KIT, LCK, SRC) | WBT (MK14) |  |
